# Bimetallic core/shell nanoparticle-decorated 3D urchin-like hierarchical TiO_2_ nanostructures with magneto-responsive and decolorization characteristics

**DOI:** 10.1186/s11671-015-0813-7

**Published:** 2015-02-27

**Authors:** Liqin Xiang, Shuo Liu, Jianbo Yin, Xiaopeng Zhao

**Affiliations:** Department of Applied Physics, Smart Materials Laboratory, Northwestern Polytechnical University, Xi’an, 710072 China

**Keywords:** Bimetallic nanoparticles, Urchin-like TiO_2_, Magnetic response, Surface plasmon resonance, Decolorization

## Abstract

The semiconductors decorated with noble metals or magnetic metals have attracted increasing attention due to multifunctional properties. In this article, we prepare novel bimetallic core/shell nanoparticle (Co@Au and Co@Ag)-decorated 3D urchin-like hierarchical TiO_2_ nanostructures through combining electroless plating and *in situ* replacement processes. The morphology and structure are characterized by scanning electron microscopy, transmission electron microscopy, energy-dispersive spectroscopy, and a surface area analyzer. It demonstrates that Co@Au and Co@Ag nanoparticles are uniformly decorated on urchin-like TiO_2_ nanostructures. The composite nanostructures show not only surface plasmon absorption band from Au or Ag but also a magneto-responsive characteristic from Co. This allows composite nanostructures to exhibit advantages including enhanced decolorization efficiency compared to pure TiO_2_ nanostructures and facile separation from a solution by magnetic field.

## Background

The combinations of semiconductors with noble metals or magnet metals have been demonstrated to be interesting in fields ranging from catalysis and optics to biotechnology [1]. Besides doping noble metals or magnet metals into semiconductors, the assembly of noble metal or magnetic metal nanoparticles onto semiconductors with special morphology, such as nanospheres, tubes and wires, films, and even three-dimensional nanostructures, has raised great interest [2,3]. Different from doped systems, the composite nanostructures may combine the unique properties of semiconductors and noble metals or magnetic metals and show multifunctional behaviors [2,3].

Titania (TiO_2_) is a high-performance functional material with a wide range of applications due to its semiconductive and biocompatible natures [4]. TiO_2_ decorated with nanoparticles of noble metal has attracted enormous attention. However, the simultaneous and effective control of the composition, morphology, structure, and distribution of decorated nanoparticles is still a challenge. In recent years, a lot of effort has been made on the study of TiO_2_ microspheres decorated with monometallic nanoparticles. And even there are a few reports about 3D hierarchical TiO_2_ microspheres decorated with noble metal nanoparticles. In the previous work, we have prepared a kind of Au or Ag nanoparticle-decorated 3D urchin-like TiO_2_ nanostructures, which exhibit an enhanced photocatalytic activity compared with the pure urchin-like TiO_2_ nanostructures and commercial P25 photocatalyst [5]. However, the percentages of methyl blue degraded by Ag-decorated TiO_2_ and Au-decorated TiO_2_ are only about 78% and 68% after 3 h of UV–vis light irradiation [5]. So, there is still a large space to improve on the degraded efficiency.

Bimetallic nanostructures often exhibit improved physical and chemical properties over their monometallic counterpart and hence are useful in many applications [6]. Especially, core/shell nanoparticles with a magnetic core and a noble-metallic shell have attracted a great deal of attention [7]. The magnetic core can provide magnetic functionality and delivery power and the noble-metallic shell can offer a well-developed function, such as surface for biomolecule attachment and plasmonically active components for optical imaging [8]. Core/shell nanoparticles, such as Fe_3_O_4_/Au, Co/Ag, and Co@Au, have attracted considerable attention [9-11]. These materials have great potential applications in the areas of electronics, photonics, catalysis, biotechnology, and so on.

Herein, we report novel 3D hierarchical TiO_2_ nanostructures decorated with bimetallic nanoparticles that have a magnetic cobalt core and a silver or gold nanoshell. The samples are prepared through a two-step method involving electroless plating and *in situ* replacement processes. The resulting composite 3D nanostructures show a distinct surface plasmon absorption band in the visible region and a good magnetic response to an external magnetic field. Furthermore, the composite 3D nanostructures can quickly decolorize methyl blue solution at room temperature. The bimetallic nature and 3D hierarchical architecture have shown excellent properties in extensive application. The combination of such bimetallic nanoparticles and TiO_2_ with a hierarchical nanostructure can not only combine multiple functionalities of dissimilar materials but also address the aggregation of nanoparticles. To the best of our knowledge, there is no report of the synthesis of the magnetically bimetallic core-shell nanospheres-decorated 3D hierarchical TiO_2_ nanostructures. We present here the preparation, characterization, and the properties of two kinds of magnetically bimetallic core-shell nanoparticles (Co@Au and Co@Ag)-decorated 3D urchin-like hierarchical TiO_2_ nanostructures in detail.

## Methods

### Synthesis of hierarchical TiO_2_ nanostructures

Hierarchical TiO_2_ nanostructures were obtained by a solvothermal method described in our previous article [12,13]. In a typical synthesis, tributyltin chloride (TBT) was dissolved in toluene in an ice-water bath, and subsequently, TiCl_4_ aqueous solution was added dropwise into the TBT/toluene solution under stirring. The mixture was transferred into a stainless steel autoclave lined with Teflon and heated at 150°C for 24 h. The precipitates were filtered, washed with ethanol several times, and dried to obtain urchin-like 3D hierarchical TiO_2_ nanostructures.

### Pre-activation of hierarchical TiO_2_ nanostructures

In order to decorate with metal nanoparticles, TiO_2_ nanostructures were pre-activated by the method as described in Ref. [14]. Firstly, the TiO_2_ particles were dispersed in a solution of 0.1 M SnCl_2_/0.1 M HCl for 40 min. During this process, the ‘sensitizer’ (Sn^2+^) was modified onto the surface of TiO_2_ nanostructures. Then, the particles were washed with water and centrifuged, and the supernant was discarded. Subsequently, the Sn^2+^-sensitized TiO_2_ nanostructures were dispersed in an aqueous solution of 1.5 × 10^−3^ M PdCl_2_/0.25 M HCl for another 40 min. During this process, the ‘catalyzer’ (Pd crystal seeds) was deposited onto the surface of TiO_2_ nanostructures. Finally, the particles were washed with water again by centrifugation to get Pd-modified TiO_2_ nanostructures.

### Synthesis of Co nanoparticle-decorated hierarchical TiO_2_ nanostructures

An amount of 0.5 g of activated TiO_2_ particles was dispersed in an aqueous CoCl_2_ solution (50 mL, 0.01 to 0.025 M) by mechanical stirring. Then, the suspension was protected by N_2_ and placed in a water bath at 30°C. After stirring for 1 h, a freshly prepared and ice-cold NaBH_4_ solution (80 mL, 0.005 to 0.015 M) was added dropwise with the rate of 2 mL/min. Within several minutes, the color of suspension changed from pink to dark gray, indicating the formation of Co nanoparticles. After further reaction for 40 min under stirring, the precipitate was separated by a magnet and washed with water three times to get Co nanoparticle-decorated TiO_2_ nanostructures. They are named as Co/TiO_2_.

### Synthesis of Co@Au or Co@Ag nanoparticle-decorated hierarchical TiO_2_ nanostructures

Co@Au or Co@Ag nanoparticles are facilely prepared at room temperature by *in situ* replacement reaction, in which Co nanoparticles are partly replaced by a noble metal salt. The process is shown as follows: 0.2 g of Co nanoparticle-decorated TiO_2_ nanostructures were dispersed into an aqueous HAuCl_4_ solution (50 mL, 0.5× 10^−3^ M) or AgNO_3_ solution (50 mL, 1.5 × 10^−3^ M) by mechanical stirring. After stirring for 1 h, the precipitate was centrifuged and washed with water three times to get resulting Co@Au or Co@Ag nanoparticles-decorated hierarchical TiO_2_ nanostructures. They are named as Co@Au/TiO_2_ or Co@Ag/TiO_2_.

### Characterization

The morphology of samples was observed by scanning electron microscopy (SEM; JSM-6700 F, Electron Optics Laboratory Co., Ltd, Tokyo, Japan) and transmission electron microscopy (TEM; JEOL-3010, Electron Optics Laboratory Co., Ltd, Tokyo, Japan). The chemical composition of samples was analyzed by transmission electron microscopy equipped with energy-dispersive X-ray (TEM/EDX; JEOL-3010, Electron Optics Laboratory Co., Ltd, Tokyo, Japan) spectroscopy. The special surface areas were determined by a Quantachrome Nova2000e surface area and pore size analyzer (Quantachrome Instruments, Boynton Beach, FL, USA). Absorbance spectra were measured using UV–vis spectrophotometer (HITACHI U-4100, HITACHI High-Technologies Corporation, Tokyo, Japan).

### Decolorization procedures

Analytical-grade methyl blue (MB, molecular formula: C_37_H_27_N_3_Na_2_O_9_S_3_, supplier: Tianjin Chemical Reagent Co. Ltd of China) was served as the target dye. Decolorization experiments are conducted at room temperature with a prepared solution of 40 mg/L MB dye in a 100-mL beaker. Typically, 10 mg of pure urchin-like TiO_2_, Co@Au/TiO_2_ or Co@Ag/TiO_2_ were respectively added into 30 mL of MB aqueous solution. After the suspension was stirred for about 15 min under daylight lamp of 40 W, the catalyst was separated by centrifugation and the reaction mixture was analyzed by UV–vis spectrophotometer (HITACHI U-4100).

## Results and discussion

**Figure 1:**
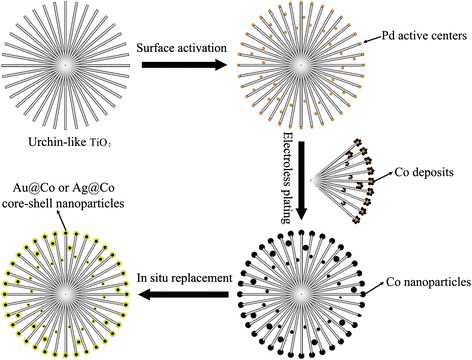
**Synthesis process of the 3D urchin-like hierarchical TiO**_**2**_ **nanostructures decorated with magnetically bimetallic core-shell nanostructures.**

The synthesis process of the 3D urchin-like hierarchical Co@Au/TiO_2_ or Co@Ag/TiO_2_ nanostructures is shown schematically in Figure 1. Firstly, the urchin-like hierarchical TiO_2_ nanostructures with diameters 1 to 4 μm are obtained by a solvothermal method [12,13]. Secondly, the surface of the urchin-like TiO_2_ is implanted with Pd nanodots by activating treatment. The Pd nanodots will act as a catalyst for the next electroless plating of Co nanoparticles. Thirdly, Co nanoparticles are deposited around the Pd active centers in an electroless plating solution to obtain Co/TiO_2_. Finally, the Co/TiO_2_ particles are dispersed into AgNO_3_ solution or HAuCl_4_ solution. Owing to the presence of seed Co nanodots, Ag^+^ or Au^3+^ is reduced and Ag or Au shell is deposited outside the Co nanodots. Thus, 3D urchin-like hierarchical Co@Au/TiO_2_ or Co@Ag/TiO_2_ nanostructures are formed by *in situ* replacement.

**Figure 2:**
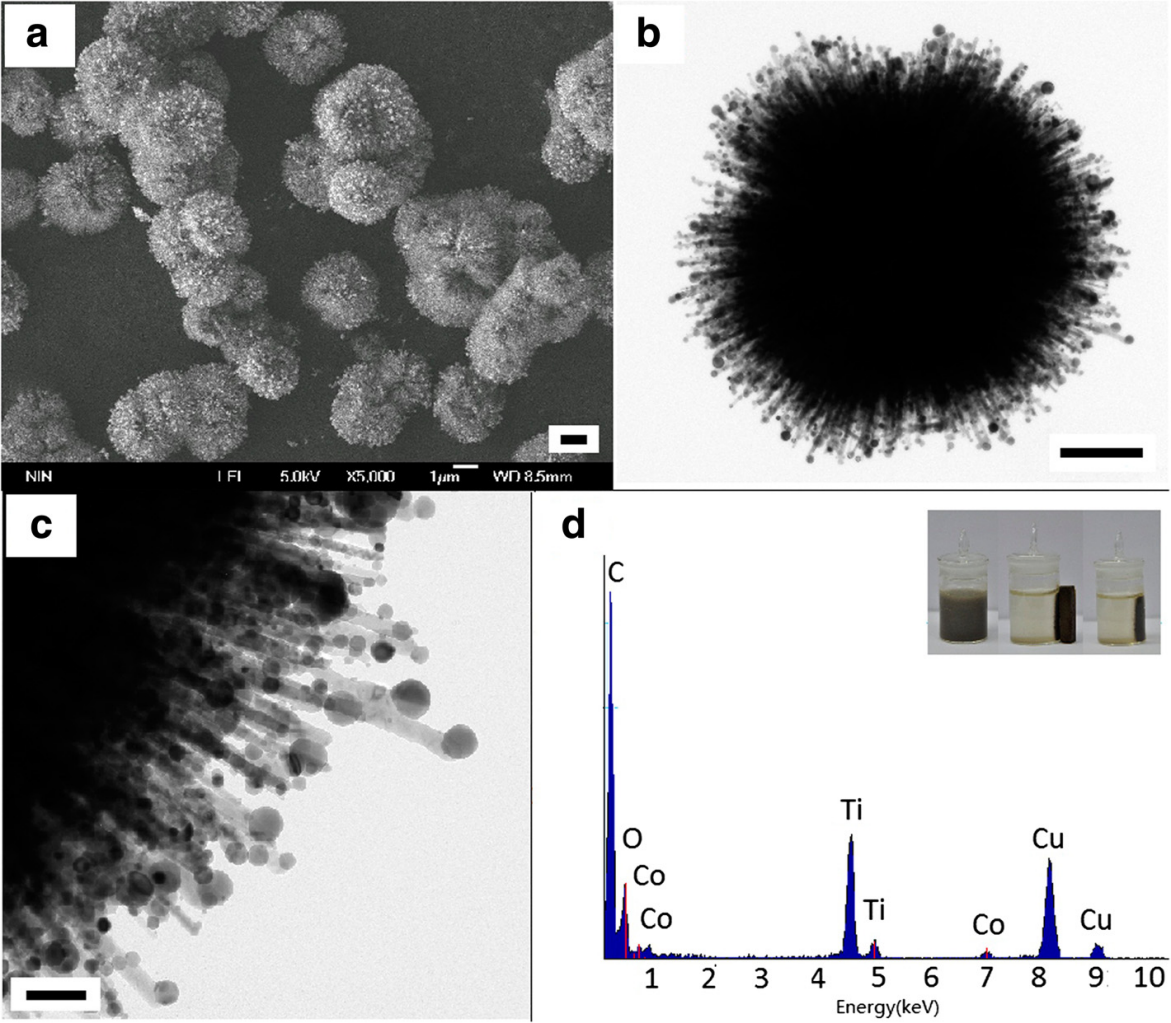
**SEM image (a), TEM images (b, c), and EDX spectra (d) of the hierarchical Co/TiO**_**2**_ **nanostructures.** Inset in **(d)** shows the response of Co/TiO_2_ to a magnet. (Scale bar = 1 μm for **(a)**, scale bar = 500 nm for **(b)**, scale bar = 100 nm for **(c)**).

**Figure 3:**
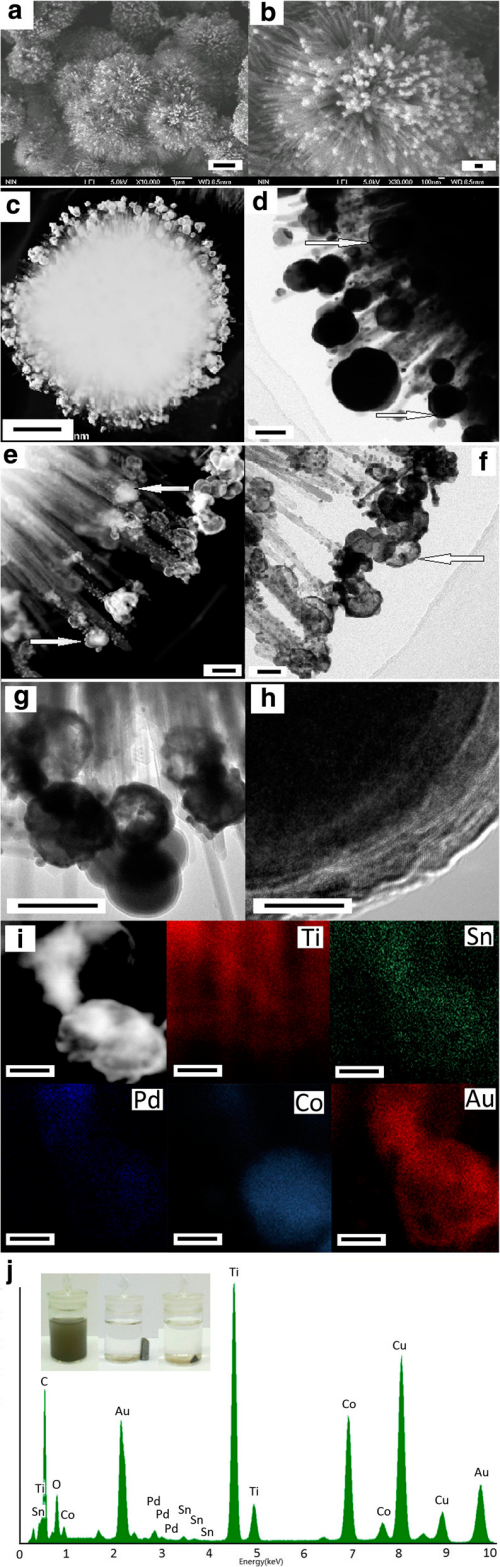
**Typical morphology, structure, and composition of the hierarchical Co@Au/TiO**_**2**_ **nanostructures.** SEM images **(a, b)**, dark-field and bright-field TEM images **(c-f)**, high-resolution TEM images **(g, h)**, the local elemental mapping **(i)**, and corresponding EDX spectra of the nanoparticles on the tips of TiO_2_ nanorods **(j)** for the 3D urchin-like hierarchical Au@Co/TiO_2_ nanostructures. The inset in **(j)** shows the response of Au@Co/TiO_2_ to a magnet. (Scale bar = 1 μm for **(a)**, scale bar = 100 nm for **(b)**, scale bar = 500 nm for **(c)**, scale bar = 100 nm for** (d-g)**, scale bar = 10 nm for **(h)**, scale bar = 50 nm for **(i)**).

The morphology and structure of the pure urchin-like hierarchical TiO_2_ nanostructures have been characterized in detail in our previous article [12]. The diameters of TiO_2_ nanostructures can be well controlled in the range of 1 to 4 μm. Figure 2 shows the typical SEM and TEM images of Co/TiO_2_ nanostructures with the mole ratio of Co to TiO_2_ of 0.15:1. It can be observed from Figure 2a that small spherical Co nanoparticles are assembled on the surface of 3D urchin-like TiO_2_. According to the high-resolution TEM images as shown in Figure 2b, c, the Co nanoparticles are firmly attached on the surface of TiO_2_ nanorods, and their diameters are in the range of 10 to 100 nm. Furthermore, the Co nanoparticles formed on the tip of TiO_2_ nanorods are larger than that formed in the middle of nanorods. This can be ascribed to the space limitation. In addition, the size of Co nanoparticles, especially on the tip of TiO_2_ nanorods, increases with the ratio of Co to TiO_2_. The EDX analysis in Figure 2d shows that the Co/TiO_2_ is composed of Ti, Co, and O elements. The signals of Cu and C are from the copper grid used. No Sn and Pd elements are detected by the EDX analysis, which may be due to their low content. However, small amounts of Sn and Pd elements can be determined by local high-resolution element mapping characterization (see Figure 3). The as-synthesized Co/TiO_2_ sample shows a good magnetic response when exposed to an external magnetic field, as shown by the photograph inset in Figure 2d. These indicate that the Co nanoparticles have been well decorated onto the hierarchical TiO_2_ nanostructures.

Figure 3 shows the typical morphology, structure, and composition of the Co@Au/TiO_2_ nanostructures that are prepared with the mole ratio of HAuCl_4_:CoCl_2_:TiO_2_ = 0.015:0.15:1. The SEM image in Figure 3a, b shows that the nanoparticles with an average diameter of about 80 nm are uniformly covered on the tips of TiO_2_ nanorods, and the morphology of the Co@Au/TiO_2_ particles looks like fireworks in full bloom. Both dark-field TEM image in Figure 3c and bright-field TEM image in Figure 3d show that most of the nanoparticles attached on tips of TiO_2_ nanorods are spherical. The high-resolution bright and dark TEM images in Figure 3d, e, f, g, h clearly show that most of the nanoparticles are core-shell nanostructures as indicated by arrows in the figures. Meanwhile, some nanoparticles are well-defined core-shell structures as shown by arrows in Figure 3d, e, while some nanoparticles are complete hollow shell structures as shown by arrows in Figure 3f. In addition, there are many nanoparticles with diameters less than 10 nm adsorbed on the side of nanorods as shown in Figure 3e, f. These different structures of nanoparticles tell us that the replacement process is complex. The Co nanoparticles attached on the tip of nanorods have more opportunities to react with Au^3+^, and they are replaced more absolutely, and, as a result, the hollow Au shells are formed [15]. However, the Co nanoparticles attached on the middle sites of nanorods have few chances to contact with Au^3+^ due to space limitation, and they are replaced incompletely, and, as a result, core/shell structures are not formed. In order to identify the bimetallic core-shell structure, the composition and structure of nanoparticles on the surface or tips of TiO_2_ nanorods are further characterized by high-resolution TEM and the elemental mapping as shown in Figure 3g, h, i. It can be found from Figure 3g that the Co@Au nanoparticles are core/shell structures but they are not uniform. Some ones are well-defined core-shell structures, some are hollow, while some solid nanoparticles have a very thin shell. These different structures of nanoparticles indicate that the replacement process is complex. The local element mapping of this region shows the Co@Au/TiO_2_ mainly contains Ti, Sn, Pd, Co, and Au. Meanwhile, the nanorods are mainly made of Ti element and small amounts of Sn and Pd elements. The nanoparticle on the tip is composed of Co and Au elements, and small amounts of Sn and Pd elements. In particular, the Co and Au distribution clearly shows the core/shell structure, which is in accordance with the TEM observation in bright-field and dark-field. Figure 3j gives the EDX analysis of this local element mapping region. The content of Ti, Sn, Pd, Co, and Au is 47.39, 0.80, 1.62, 28.55, and 21.53 at%, respectively. On the basis of these characterizations, it can be identified that hierarchical TiO_2_ nanostructures decorated with core-shell Co@Au nanoparticles have been successfully prepared. The resulting Co@Au/TiO_2_ also shows a good magnetic response when exposed to an applied external magnetic field as shown by the photograph inset in Figure 3j.

**Figure 4:**
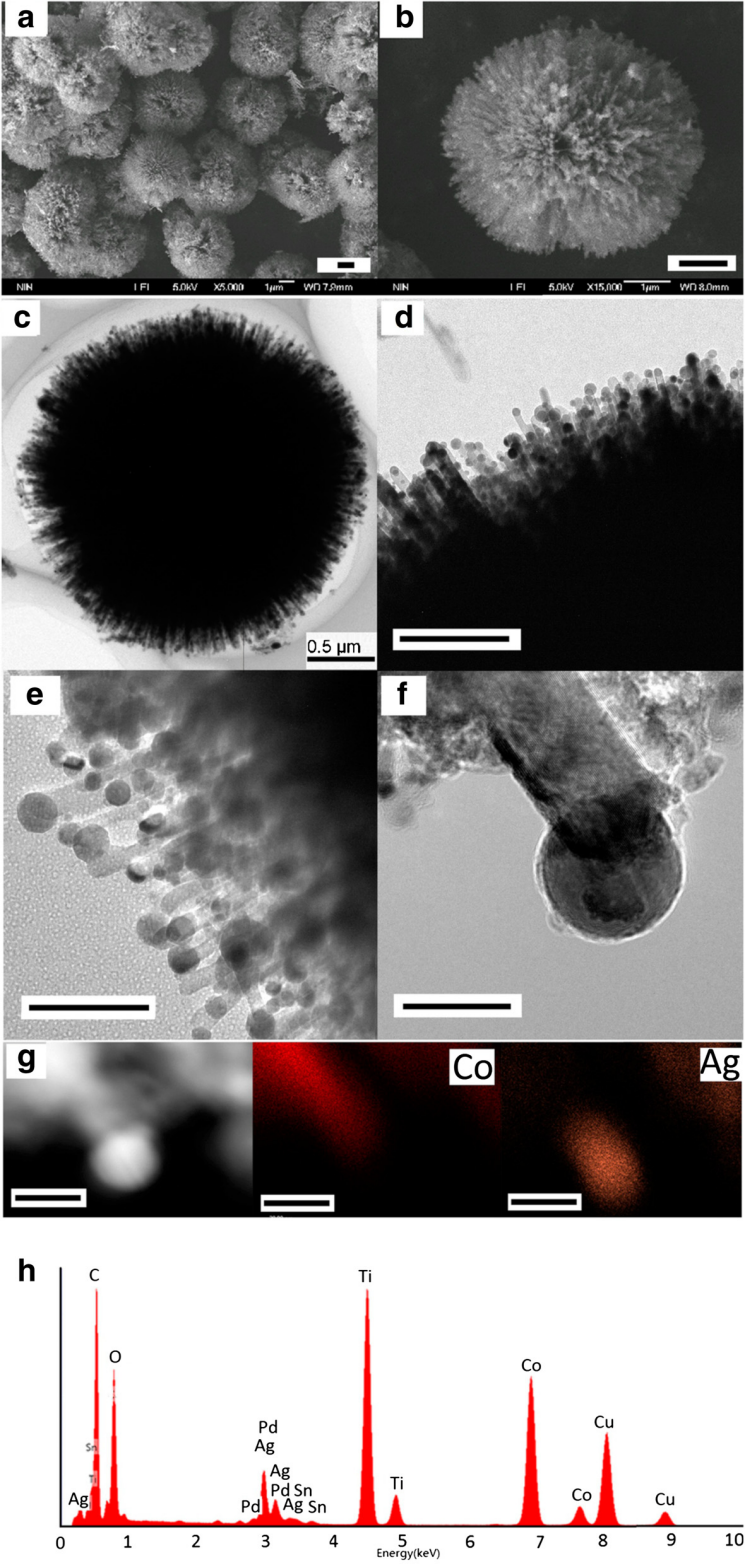
**Typical morphology, structure, and composition of the hierarchical Co@Ag/TiO**_**2**_ **.** SEM images **(a, b)**, TEM images **(c, d)**, and high-resolution TEM images **(e, f)**, the local elemental mapping **(g)**, and corresponding EDX spectra of the nanoparticles on the tips of TiO_2_ nanorods **(h)** for the 3D urchin-like hierarchical Ag@Co/TiO_2_ nanostructures. (Scale bar = 1 μm for **(a-b)**; scale bar = 500 nm for **(c)**; scale bar =200 nm for **(d)**; scale bar = 100 nm for **(e)**; scale bar = 20 nm for **(f-g)**).

Figure 4 shows the morphology and the structure of a typical sample of Co@Ag /TiO_2_ with a mole ratio of AgNO_3_:CoCl_2_:TiO_2_ = 0.03:0.1:1. The SEM images (Figure 4a, b) show many light nanodots are adsorbed on urchin-like TiO_2_ microspheres with the diameters about 3 μm. The TEM images (Figure 4c, d, e, f) show the nanodots cover on TiO_2_ nanorods homogeneously, and the average diameters of the nanodots are about 10 to 20 nm. The composition and structure of nanoparticles on the tips of TiO_2_ nanorods are mainly composed of Ti, Sn, Pd, Co, and Ag elements as shown in Figure 4g, h. The content of Ti, Sn, Pd, Co, and Ag is 48.16, 1.46, 1.15, 36.96, and 12.27 at%, respectively.

**Figure 5:**
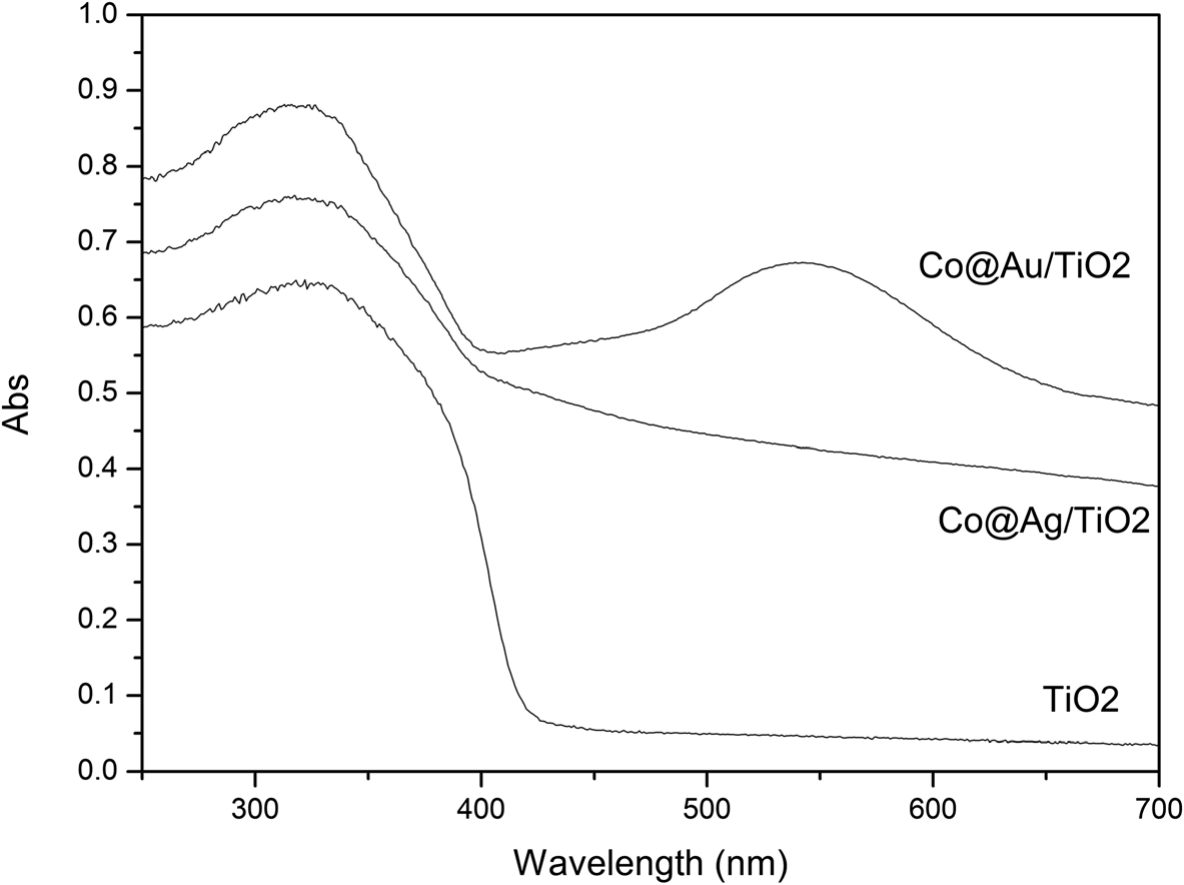
**UV-visible absorption spectra of the hierarchical TiO**_**2**_ **, Co@Au/TiO**_**2**_ **, and Co@Ag/TiO**_**2**_ **nanostructures.**

The UV–vis absorption spectra of the urchin-like hierarchical TiO_2_, Co@Au/TiO_2_, and Co@Ag/TiO_2_ nanostructures are showed in Figure 5. Obviously, decorating with Co@Au or Co@Ag bimetallic nanoparticles can enhance light-harvesting efficiency. Besides the absorption band of TiO_2_ at the wavelength lower than 400 nm, a wide absorption band in the range of visible light can be observed in the spectra of Co@Au/TiO_2_ and Co@Ag/TiO_2_ nanostructures. Especially, the Co@Au/TiO_2_ nanoparticles show a strong absorption peak at about 550 nm corresponding to the surface plasmon resonance of Au nanoparticles. The Co@Ag/TiO_2_ nanostructures do not show a strong absorption peak mainly because the corresponding surface plasmon resonance of Ag nanoparticles is located at a lower wavelength than 400 nm.

**Figure 6:**
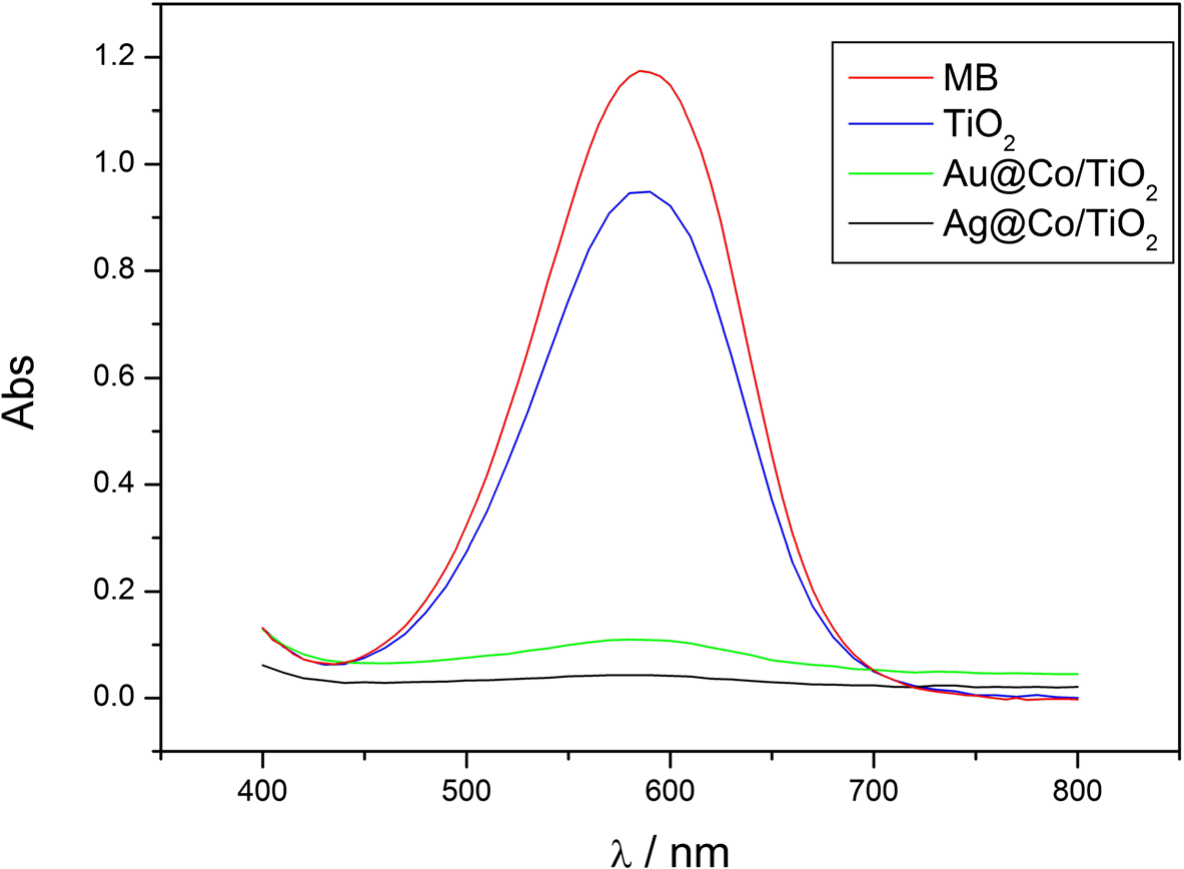
**UV-visible absorption spectra of MB after interaction with hierarchical TiO**_**2**_ **, Co@Au/TiO**_**2**_ **, and Co@Ag/TiO**_**2**_ **nanostructures.** The spectra were measured after daylight lamp irradiation for 15 min.

Finally, the Co@Au/TiO_2_ and Co@Ag/TiO_2_ nanostructures are also evaluated for decolorization of methyl blue (MB) solution, a model dye in wastewater of the dyeing industry. The removal of MB molecules from the aqueous solution using Co@Au/TiO_2_ and Co@Ag/TiO_2_ nanostructures is monitored by UV–vis spectroscopy as shown in Figure 6. It is found that the intensity of the characteristic absorption peak of MB at 590 nm significantly decreases in the presence of Co@Au/TiO_2_ or Co@Ag/TiO_2_ nanostructures, indicating the rapid decolorization of MB. In particular, compared to urchin-like TiO_2_ nanostructures, the Co@Au/TiO_2_ or Co@Ag/TiO_2_ nanostructures show faster decolorization rate. For example, after daylight lamp irradiation for 15 min, the decrease of peak intensity of MB at 590 nm exceeds 90% in the presence of Co@Au/TiO_2_ nanostructures and exceeds 95% in the presence of Co@Ag/TiO_2_ nanostructures, which is distinctly faster than 19% of decolorization by pure urchin-like TiO_2_. According to the N_2_ adsorption-desorption isotherm, the special surface area of pure TiO_2_, Co@Au/TiO_2_, and Co@Ag/TiO_2_ nanostructures is 35, 31, and 34 m^2^/g, respectively. The difference in the surface area of three samples is not significant. Therefore, the faster decolorization of MB solution in the presence of Co@Au/TiO_2_ and Co@Ag/TiO_2_ nanostructures can be mainly attributed to Co@Au and Co@Ag nanoparticles decorated on TiO_2_, which have enhanced light-harvesting ability and improved the photocatalytical efficiency. The Co@Au/TiO_2_ and Co@Ag/TiO_2_ nanostructures can also retain their activity in repeated decolorization cycles. For example, after cycling decolorization for five times, the typical Co@Au/TiO_2_ nanostructures still maintain 85% of decolorization after irradiation for 15 min, while Co@Ag/TiO_2_ nanostructures maintain 90% of decolorization. In addition, it should be noted that the Co@Au/TiO_2_ or Co@Ag/TiO_2_ is also suitable for decolorization of other dyes, such as methyl orange (MO), etc., but the decolorization rate for MO is much slower compared to that for MB. The similar phenomenon has also been reported in other references, which can be attributed to the difference of molecular structure between MO and MB [16].

## Conclusions

In this paper, we have presented a type of 3D urchin-like hierarchical TiO_2_ nanostructures decorated with magnetically bimetallic core-shell nanoparticles. The urchin-like TiO_2_ nanostructures are used as templates, magnetic Co nanoparticles are introduced onto TiO_2_ nanostructures by electroless plating, and finally, the Au or Ag shell is formed on Co nanoparticles by *in situ* replacement reaction. The resulting Co@Au/TiO_2_ and Co@Ag/TiO_2_ nanostructures not only possess a good magnetic response to an applied external magnetic field but also show enhanced light-absorption ability due to surface plasmon absorption of Au and Ag in the visible region. Decolorization experiments show that the Co@Au/TiO_2_ and Co@Ag/TiO_2_ nanostructures can decolorize methyl blue more effectively compared to pure TiO_2_ nanostructures. The unique structures and enhanced multifunctional characteristics will allow the 3D bimetallic core-shell nanoparticle-decorated TiO_2_ nanostructures to have potential applications in a wide range of fields.
